# ﻿Construction of the fecal armor of larvae of *Podontia quatuordecimpunctata* (L.) (Chrysomelidae, Galerucinae, Alticini) and its role against insecticides in pest management

**DOI:** 10.3897/zookeys.1252.151737

**Published:** 2025-09-19

**Authors:** Orlando A. Calcetas, Joel L. Adorada, Michael Schmitt, Caroline S. Chaboo

**Affiliations:** 1 Department of Agriculture, Regional Field Office No. 4A-CALABARZON, Regional Crop Protection Center (DA-RCPC-IVA), Lipa Agricultural Research and Experiment Station (LARES), Brgy. Marawoy, Lipa City, Batangas, 4217, Philippines Lipa Agricultural Research and Experiment Station Lipa City Philippines; 2 Bureau of Plant Industry-Los Baños National Crop Research, Development and Production Support Center (BPI-LBNCRDPSC), Timugan, Los Baños, Laguna, 4030, Philippines Bureau of Plant Industry-Los Baños National Crop Research, Development and Production Support Center Los Baños Philippines; 3 Universitaet Greifswald, Allgemeine & Systematische Zoologie Loitzer Str. 26, D-17489, Greifswald, Germany Universitaet Greifswald Greifswald Germany; 4 Division of Entomology, University of Nebraska State Museum, University of Nebraska-Lincoln, 306 Morrill Hall, Lincoln, NE 68588-0338, USA University of Nebraska State Museum, University of Nebraska-Lincoln Lincoln United States of America

**Keywords:** Architecture, defenses, behaviors, predators, *
Spondias
*

## Abstract

Five subfamilies within Chrysomelidae (leaf beetles) have larvae that retain their feces as a coat or armor which serves for thermoregulation, camouflage, or barrier to enemies. The construction, retention and repair of these fecal structures are associated with specialized larval morphologies in the tortoise beetles (subfamily Cassidinae) and in the Cryptocephalinae + Lamprosomatinae (Camptosomata), but morphology associated with fecal encrustations on larvae in the *Blepharida*-group flea beetles (Galerucinae: Alticini) and in Criocerinae have not been examined. Experiments with live larvae of *Podontia quatuordecimpunctata* (L., 1767) (or sineguelas leaf beetle, SLB; *Blepharida* group) reveal the anus opens dorsally and deposits feces directly to the larva’s dorsum; the armor is maintained and is reconstructed. Scanning electron microscopy reveals integumental microtrichia that presumably hold on the feces. This invasive beetle has become an introduced tree-crop pest in the Philippines, so ongoing research seeks to mitigate its population. Insecticidal chemical assays show that fecal armor does not fully protect SLB larvae but delays potency slightly. The study recommends rotating the insecticides (Imidacloprid, Cypermethrin, and Buprofezin) to prevent the development of resistance. Specialized morphology for fecal retention is known in Cassidinae, Camptosomata and is now documented in the *Blepharida* group. Such morphology and the fecal-building behavior can offer additional phylogenetic information for these beetles.

## ﻿Introduction

Many animals are known to build structures that serve for protection and as nurseries, domiciles, and traps (von Frisch 1974; Hansell 2005). Darwin (1859: 224–235; 1881 (letter)) even discussed the building behavior in wasps. Five groups within Chrysomelidae (leaf beetles) have larvae that shape their feces into a coat, case or shield which serve for thermoregulation, camouflage, or offense/deterrent to enemies (Olmstead and Denno 1992; Morton and Vencl 1998; Nogueira-de-Sá and Trigo 2002; Müller and Hilker 2003; Gómez 2004; Bacher and Luder 2005; Chaboo et al. 2008; Huang et al. 2022). Chaboo et al. (2023) reviewed various constructions built by many insects and reported on shield constructing behaviors and associated morphology in tortoise beetle larvae (~3000 species in 10 derived tribes of Chrysomelidae: Cassidinae (Chaboo 2007)). The other chrysomelid fecal builders are the large radiation of (Cryptocephalinae + Lamprosomatinae) (= Camptosomata; ~6000 species) and two smaller lineages, Criocerinae (~1400 species) and the *Blepharida* group (~200 species; Galerucinae: Alticini).

*Podontia quatuordecimpunctata* (SLB) is being studied in the Philippines where it was introduced from south-east Asia and has become a defoliating pest of *Spondias purpurea* Linnaeus, 1762 (Anacardiaceae; sineguelas tree), a fruit tree introduced from the Neotropics. Author Calcetas has led research on SLB and published on its economic impact (Adorada et al. 2023), SLB biology (Calcetas et al. 2023), management strategies (Calcetas et al. 2024), and tree biology (unpubl. data). *Podontia quatuordecimpunctata* is now called sineguelas leaf beetle (SLB) in the Philippines to ease communication about this pest.

Research on chrysomelid fecal structures reveals specialized morphology for construction, retention and repair that reflects broader phylogenetic patterns. In tortoise beetles (= ten “derived” tribes of Cassidinae), larvae use a telescopic anus to attach their feces into caudal processes (urogomphi). Exuviae may be retained at each molt and the combined exuvio-fecal shield is held together by inter-nested processes (Chaboo et al. 2023); this shield may be inherited by pupae. In Cryptocephalinae and Lamprosomatinae, the swollen larval abdomen acts as a “plug” that holds the fecal case like an oversized hat over the larva; this hard protective case also serves as the pupation chamber (Brown and Funk 2005; Chaboo et al. 2008). In the *Blepharida*-group flea-beetles and in Criocerinae larvae have a dorsal anus and excreted fecal pellets coat the dorsum as they move towards the head. The feces are held directly on the body in contrast to the discrete structures in Cassidinae and Camptosomata. The process of fecal construction has been reported for some species of Cassidinae (Chaboo et al. 2023) and Cryptocephalinae (Brown and Funk 2005). We presume the process and specialized morphology in Cryptocephalinae is similar in the sister subfamily, Lamprosomatinae.

Fecal construction and retention in *Blepharida*-group flea beetles and in Criocerinae are not well studied. Around 22 genera comprise the *Blepharida*-group flea beetle lineage, well-defined morphologically within Alticini by adult features (Pramanik and Basu 1973; Furth 1992; Medvedev 1999; Becerra 2004; Prathapan and Chaboo 2011; Biondi et al. 2017; D’Alessandro and Biondi 2023, 2025) and by the larvae which have the dorsal anus, placing feces directly on the dorsum (Paterson 1943). Prathapan and Chaboo (2011) cleaned larvae of *Podontia congregata* Baly, 1865 (*Blepharida* group) with a camel-hair brush and followed reconstruction of the fecal coat over 6–8 h. Vencl and Morton (1998) examined one species (*Blepharida* group) and inferred a special neuro-muscular propulsion that may move feces from the caudal anus towards the head. The defensive role of fecal armor against insect enemies (e.g., ants, bugs, parasites) was investigated experimentally in just two species, one of *Blepharida* Chevrolat, 1836 (Vencl and Morton 1998) and other of *Ophrida* Chapuis, 1875 (Huang et al. 2022). Results were contradictory, showing that feces provide physical and chemical deterrence but also attract certain arthropod enemies.

**Figures 1–5.**
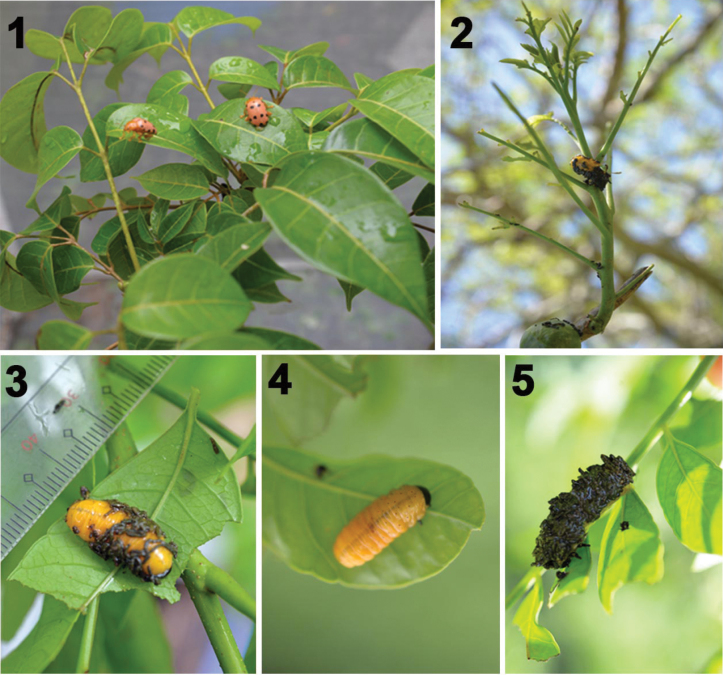
*Podontia quatuordecimpunctata* (L., 1767) on its host plant, *Spondias purpurea* L., 1762 (Anacardiaceae) in the Philippines (photos by O. Calcetas). 1. Adults; 2. Larvae defoliate the host; 3. Instar III larva with partial fecal cover; 4. Instar IV larva without fecal cover; 5. Instar IV larva with complete fecal cover.

Calcetas et al. (2023) reported on the biology of *Podontia quatuordecimpunctata* (Linnaeus, 1767), commonly called sineguelas leaf beetle (=SLB; Galerucinae: Alticini: *Blepharida* group; Fig. 1). This species has four larval instars. Each teneral instar is naked but becomes covered with black feces over the next 24 h (Figs 2–5). The SLB larval fecal armor consists of fecal plant material, partly digested and undigested fragments of leaf veins and midrib, and a sticky clear fecal fluid. Here, we investigate three aspects of this fecal armor: 1) the construction, 2) scanning electron microscopy (SEM) study of the larva’s integument for special morphological features associated with the fecal coat, and 3) the effect of insecticides to overcome this pest’s fecal armor.

## ﻿Materials and methods

For the current study, Calcetas and Adorada studied SLB larvae from greenhouse populations. Author Schmitt conducted the scanning electron microscopy (SEM) study on a sample of larvae now vouchered at University of Greifswald, Germany.

Question 1. Materials of the armor? We observed 100 specimens of SLB larvae of all instars in multiple wild populations and in our greenhouse populations. The armor was studied with the naked eye and under microscope and probed with forceps to determine the macro-components (not chemicals).

Question 2. How is the fecal armor constructed? We conducted fecal removal experiments with SLB larvae of instars II, III, and IV to determine how the armor is re-constructed and to determine a Fecal Construction Rate (FCR), how fast the larva covers its integument at a particular length of time. Experiments were done in October–November 2023 (*n* = 9 per instar, reared) and repeated in June 2024 using 38 larvae collected 30 May 2024 in Pinamukhan, Batangas City, and transferred to a 3-yr old sineguelas tree that was transplanted in San Roque, Victoria, Laguna, Philippines. These larvae were introduced on the new tree on 02–09 June 2024.

Each SLB beetle larva was placed on the adaxial surface of a sineguelas leaf, one per leaf per stem to ensure separation. Each sample shoot was numbered on the abaxial surface of the leaf; we used a Stabilo pen whose marks are not washed off by rainwater. Markings also minimize transfer and mix up of samples. Larvae were placed on the other tree on 01 June 2024 and left a day to acclimate and reduce stress from handling. A note on larval stress: some larvae take a long time to stick or attach to the tree and frequently fall from the tree, so we had to repeatedly attach them. Figs 15–26 show replications with two larvae from this experiment.

Each larva’s fecal coat was removed with a small, fine camel brush at time zero (T0 hours), following the method in Prathapan and Chaboo (2011), then the fecal cover formation was observed every two hours for 24 hours. Each leaf number was photographed, followed by the respective larva with a plastic ruler showing the millimeter scale. The data were tabulated, graphed, and analyzed (Time below is given as h = hours, min = minutes, sec = seconds). These experiments were repeated on 02 June 2024 using the same larvae and sineguelas tree.

Question 3. How are feces held on the body? This aimed to identify integumental structures that may hold the fecal armor on the larva’s body. We examined reared larvae (3 × instar II, 4 × instar IV), which were killed and preserved in 70% EtOH and shipped to Schmitt. One larva of each stage was washed in distilled water and then in ethanol. These larvae were dehydrated through a graded series of EtOH, critical point dried in a Leica EM CPD300, mounted on stubs and coated with gold-palladium. These were examined and imaged with a Carl Zeiss Evo LS10 SEM. The other larvae were cleaned of their fecal debris with KOH before drying the specimens with Hexamethyldisilazane (HMDS); these were also coated with gold-palladium and studied with a field-emission SEM Carl Zeiss Supra 40VP, both SEMs at the imaging center, University of Greifswald, Germany.

Removing the denticles is technically impossible when the larvae are alive. They are soft-bodied; fixing them to shave off the setae (sensilla) and denticles would damage the larvae and not yield sound results.

**Figures 6–8.**
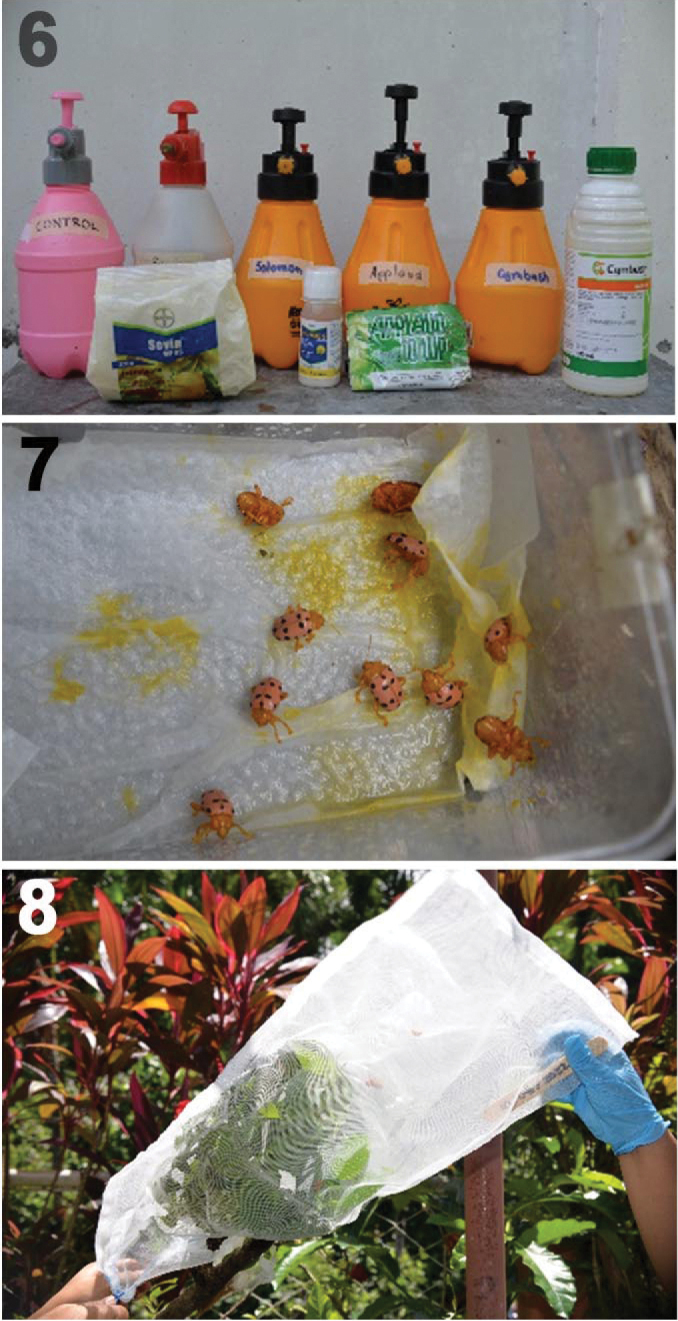
Insecticide trial set up (photos by R.A. Anabo). 6. Insecticides tested; 7. Adults sprayed with insecticide; 8. Sineguelas twig covered with nylon net.

Question 4. Does the fecal armor protect SLB from insecticides? Limited experimental work in Cassidinae (Chrysomelidae) and *Neochlamisus* Karren, 1972 (Chrysomelidae: Cryptocephalinae) demonstrated that fecal structures protect these by providing a physical, chemically enforced, distasteful barrier (see Chaboo et al. 2023 and citations therein). To examine the role of the fecal coat in SLB, we conducted experiments with insecticides (Fig. 6) in January 2024, Laguna, Philippines. The few other studies on controlling this pest used different insecticides in other countries (Uddin and Khan 2014) and fungal-based pesticides (Rahman et al. 2022).

Our tested insecticides are: Cypermethrin (a pyrethroid) – 4.15ml/1L, Carbaryl (a carbamate) – 3.75g/1L, and Imidacloprid (a neonicotinoid) – 0.4ml/1L, at concentrations based on the recommended rate of each insecticide package. These are allowed to be used in the Philippines, are commonly offered to farmers, and has low mammalian toxicity. Each solution was transferred to a 2-L hand pressure sprayer with labels written on a masking tape (Fig. 6). We used distilled water as our control. After the application of each treatment, we measured the time for each adult and larval mortality. A solution of Buprofezin, an Insect Growth Regulator (IGR), was made at the recommended concentration (1.775g/1L) and tested on different larval instars and on adults. Time to immobility, abnormal molting (molted integument still attached to the larval body), and death after treatment were recorded.

Trial with larval SLB. Two different insecticide groups, Cypermethrin (pyrethroids) and Imidacloprid (neonicotinoids), were tested on larvae raised in the greenhouse population. We did three replications (*n* = 5 each trial) with three different larval instars (II, III, IV), with and without the fecal coat (Table 1). The larvae were placed on a twig of a sineguelas seedlings before spraying directly. A 60cm x 40cm drawstring nylon net bag was also placed on the twig (Fig. 8) to prevent the larva from escaping. Immediately and up to 41 hours later, the set up was monitored. The fecal covering rate (FCR) is calculated by dividing the highest fecal cover at any given time period that it has been reached (e.g., 24 h divided by percent fecal cover).

**Table 1.** Insecticide impact on larvae (each trial *n* = 5) of *Podontia quatuordecimpunctata*, September 2023.

Insecticide		Imidacloprid	Cypermethrin
Fecal Cover	Instar	Mortality Rate	
**With**	II	100% at 6 mins, 25 secs	100% at 17 h, 40 mins
	III	100% at 17 h, 40 mins	
	IV		
**Without (Naked)**	II	100% at 5 mins, 10 secs	100% at 17 h, 40 mins
	III	100% at 17 h, 40 mins	
	IV		

Trial with adult SLB. Plastic containers with a folded paper towel and 10 adult individuals were prepared (three replications with 10 individuals per insecticide). The adults were sprayed (Fig. 7) and monitored (Table 2).

**Table 2.** Insecticide impact on adults (*n* = 10 per replication; 120 total) of *Podontia quatuordecimpunctata*, October 2023.

Treatments	Replication	Dead	Alive	Total	% Mortality	Time
**Carbaryl**	1	8	2	10	80	31 h
	2	6	4	10	60	41 h
	3	3	7	10	30	31 h
**Imidacloprid**	1	10	0	10	100	9 mins
	2	10	0	10	100	9 mins
	3	10	0	10	100	9 mins
**Cypermethrin**	1	10	0	10	100	14 mins
	2	10	0	10	100	14 mins
	3	10	0	10	100	14 mins
**Bufropezin**	1	6	0	6	100	72 h
	2	6	0	6	100	72 h
	3	6	0	6	100	72 h
**Control**	1	0	10	10	0	-
	2	0	10	10	0	-
	3	0	10	10	0	-

## ﻿Results

**Figures 9–14.**
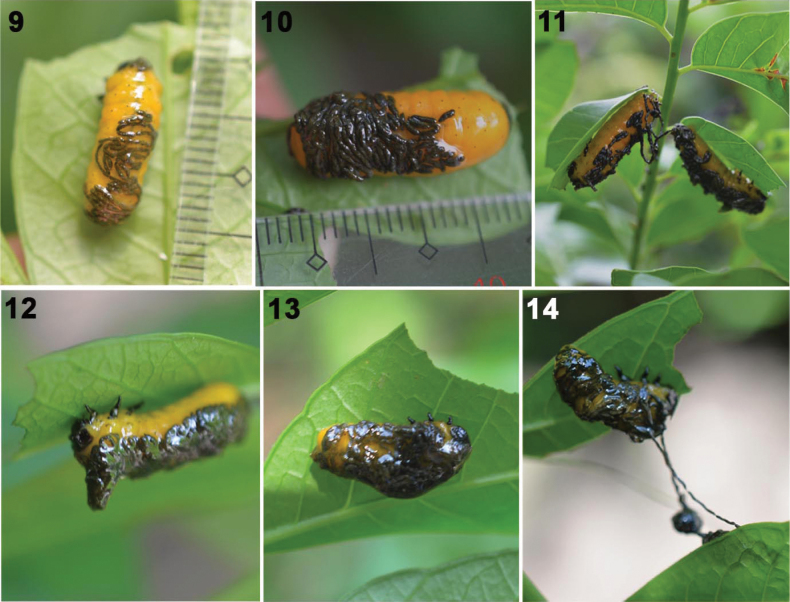
Fecal coat of larvae of *Podontia quatuordecimpunctata* (SLB) (photos by O. Calcetas and J. Adorada). 9. Instar II partially covered by its feces, pellets discernible, and excretory fluid; 10. Fecal armor thickens in dorso-posterior part of body; 11. Feces can slide haphazardly over the body; 12, 13. When larvae feed on adaxial side of leaf, feces accumulate at the head (Fig. 12) or thicken in the mid-section (Fig. 13); 14. Excretory fluids hold feces together, even when stretched out as chains.

Nature of the fecal armor (Figs 9–14). The SLB larval fecal coat appears dark yellowish when fresh and turn blackish in appearance and thread-like when dried but is dark green under microscope (Calcetas et al. 2023). The coat consists of feces and a sticky fluid; we found exuviae in the coat of larval fecal materials inside a nylon net enclosure (Calcetas et al. 2023). The clear fecal fluid may help to hold the fecal accumulation on the larval body. Sineguelas leaves lack visible trichomes and no trichomes were found in the feces. No undigested plant fragments were detected.

Fecal armor construction (Figs 9–26). After the feces are released from the dorso-posterior anus, the feces spread anteriad by peristaltic movement of the body towards the head. Fecal pellets are bacilli- (or sausage) shaped and are linked into chains by sticky body fluids (Figs 9, 10; Calcetas et al. 2023). Fecal accumulations on a larva’s body vary by gravity, feeding position, and larval movement. When the larva is not moving much, feces can accumulate on the sides, in a zig-zag pattern (Fig. 11). When in a horizontal position (Figs 12–14), feces tend to accumulate at the head (Fig. 12), likely due to the head’s upward tilt during feeding, or in the central or bent section of the body (Fig. 13).

**Figures 15–26.**
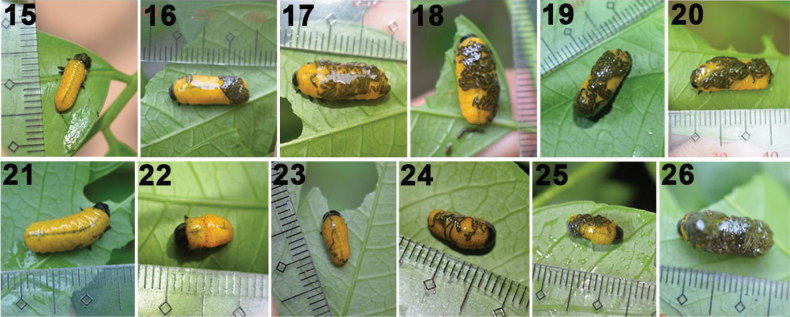
Fecal armor reconstruction over 24 hours by larvae of *Podontia quatuordecimpunctata* (photos by O. Calcetas and J. Adorada), two replicates on 20 June 2024. 15–23. Instar II larva, replication one. 15. Time 0 when larva is cleaned of fecal armor; 16. After T2 hours, a clear fecal fluid may cover the dorsum; 17. After T4 hours, a single long fecal thread and fecal fluid covers the body dorsum; 18. After T6 hours, more fecal strands cover about 50% dorsum; 19. After T8 hours, fecal strands appear layered; 20. After T24 hours, except for the head, the larva dorsum is mostly covered with fecal pellets and fluid; the pellets and strands are heaped and appear as two or three layers. 21–26. Instar IV larva, replication one. 21. Time 0 when larva is cleaned of fecal armor; 22. After T2 h, a clear fecal fluid and wet pellets cover the caudal region; 23. After T4 h, the dorsum is mostly covered with a single layer of fluid and fecal pellets; 24. After T6 h, it appears similar to T4 h, but excess pellets start falling to the substrate; 25. After T8 h, the pellets start appearing as layered; 26. After T24 h, except for the head, the larva dorsum is mostly covered with fecal pellets and fluid; the pellets appear as two or three layers.

When the larva is feeding on the abaxial surface of the leaf, this can facilitate feces falling off. However, the sticky excretory fluid helps bind the feces which form threads when stretched or pulled away from the larva’s body (Fig. 14). The bond’s strength can hold the weight of a suspended beetle. Stickiness is reduced by rainwater splashes and fecal sections may detach and scatter around the body, onto the leaf surface, or fall to the ground. Evaporation of water can also enhance stickiness and fecal thread formation. The fecal armor has varying moisture levels, from wet film to dry pellets. Many larvae can remain attached to the foliage in heavy rain and windy conditions.

We also observed in instar III–IV larvae which feed voraciously and produce large amount of feces, so that their fecal coat increases in volume. Depending on the prevailing weather, wind speed, agitation strength, larvae can retain their fecal armor. Sometimes, the feces can be dry and appear like flakes though still firmly affixed to the integument.

In October 2023 (wet season data), we found that instar II larvae (*n* = 9) can cover the entire body with feces only after an 8-h period or approximately 12.5% per hour rate while after the 24-h period they were able to cover 100% of their body with a thick coat. However, the FCR of the instar III–IV larvae coated 50% and 55% of their body respectively with a thin film of feces after eight h while after the 24-h period both larval instars were able to cover 100% of their body with a thick coat. The instar II larvae in October 2023 are not stressed and possibly are at their full potential compared to the instar II larvae in the June 2024 trial. After a 24-h period, the feces accumulate, significantly thickening the fecal coat and lumping on different body parts, depending on the larval position; some feces fall to the leaves and ground.

**Figure 27.**
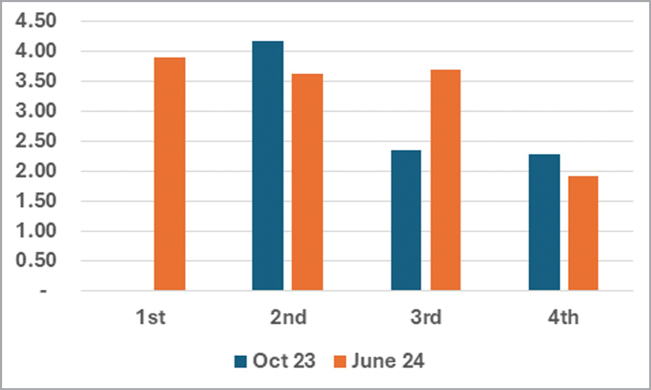
The fecal construction rate (FCR), how fast the larva covers itself in a 24-hr period, in *Podontia quatuordecimpunctata* larvae, October 2023 and June 2024 wet season trials.

**Figures 28–31.**
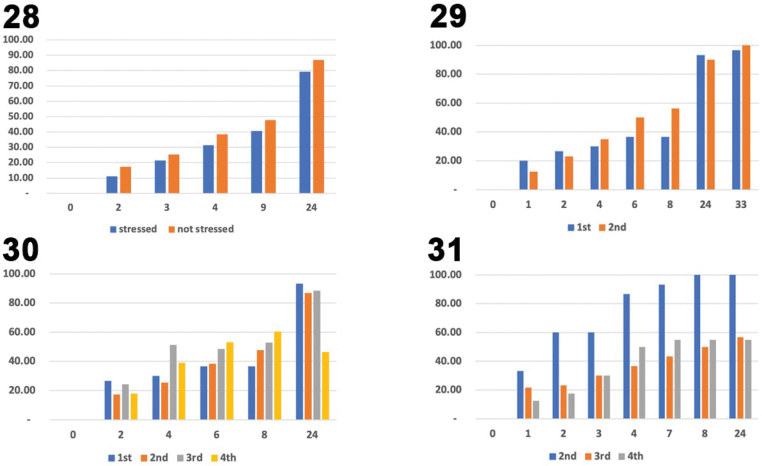
Graphs comparing larval fecal coat construction of the feeding stressed (fed on non-fresh leaves after 48-hr period) and non-feeding stressed (fed on fresh leaves after 24-hr period). Stressed and non-stressed larvae were treated as two different categories for the further analyses (see methods). x-axis = hours; y-axis = %. 28. Larva II instars of *Podontia quatuordecimpunctata* on June 2023 trial; 29. Larva I–II instars after the 24- and 33-hr period on June 2024 trial; 30. Different larval instars on June 2024 trial; 31. Different larval instars on October, 2023 trial.

Percent Fecal Construction Rate (%FCR; Figs 27–30). In the October 2023 and June 2024 data, we found that about 4.0% of all SLB larval instars covered ~50% of their body with a with a thin fecal coat after 8–9 h and nearly 100% with a thick coat after 24-h (Fig. 27). On 01–02 June2024 (Fig. 28), the FCR of SLB larvae (*n* = 15) was 40.71% after 8 h of feeding and 79.29% at 24 h. The process of individual transfer and feeding stress might contribute to rate differences. The average hourly FCR difference in a 24-h period of stressed Instar II between Oct. 2023 trial (4.17) and June 2024 trial (3.62) is 0.55 points which is not significant. Over our long monitoring, stressed larvae on the test plants seemed sluggish and less voracious compared to the Oct. 2023 individuals and to other larvae in the field. Typically, the instar II larvae are voracious and their body is always 100% covered with a fecal coat, except for those sickly ones (infected with fungus).

Data on 02–03 June 2024 (Fig. 29) show the FCR for instar I was 36.67% (*n* = 3) at the 8^th^ hr, 93.33% (*n* = 3) at the 24^th^ hr, and 96.67% (*n* = 3) 33^rd^ hr. For instar II (*n* = 8), the FCR was 56.25% after 8 h of feeding, 90.00% at 24^th^ hr, and 100.00% the 33^rd^ hr. The fecal coat at T33 h is very thick but some feces fall apart, detach from the larval body, or clump on different body sections.

Data on 06–07 June 2024 (Fig. 30) show the FCR for instar II (*n* = 6) was 56.67% at the 9^th^ hr, 90.00% at the 24^th^ hr, and 85.00% (*n* = 6) at the 33^rd^ hr (reading after a two-hr heavy rain). During this period, we documented that instar II larvae (*n* = 14) can cover the body ~3.42–3.75% with fecal material in a 24-hr period). For the instar III (*n* = 5, FCR was higher at 52.86%), 88.57% (*n* = 5) at the 24^th^ hr, and 85.00% (*n* = 5) at the 33^rd^ hr (recorded after a 2-hr heavy rain). Two long 1-hr heavy afternoon rains reduced the average fecal cover of both larval instar II and III from 89.29% to 85%.

FCR data (Fig. 30) on 08–09 June 2024 for instar IV (*n* = 12) was 60.45%, and 46.36% at the 24 hr. In this period the instar IV larva feeds less as they approached the pre-pupal stage. For instance, almost 50% (*n* = 6) already pupated while some went missing, probably falling to the ground voluntarily to pupate.

Similar results were observed on some outlier SLB larvae in the trial. Some weakened, stressed, sickly larvae fed less and have FCR? at approximately 5% fecal material excreted throughout the 8^th^ or 9^th^ day period. A few larvae were able to cover their body with a thin fecal coat after the 24^th^ hr period while others went missing or probably died. One larva was observed frequently moving in a “push-up” motion, not producing feces, with its ambulatory lobes attached securely to the leaf surface. Later, this larva died, suspended upside down by a silken thread to the leaf surface; death could be due also to bacterial or viral infection.

**Figures 32–37.**
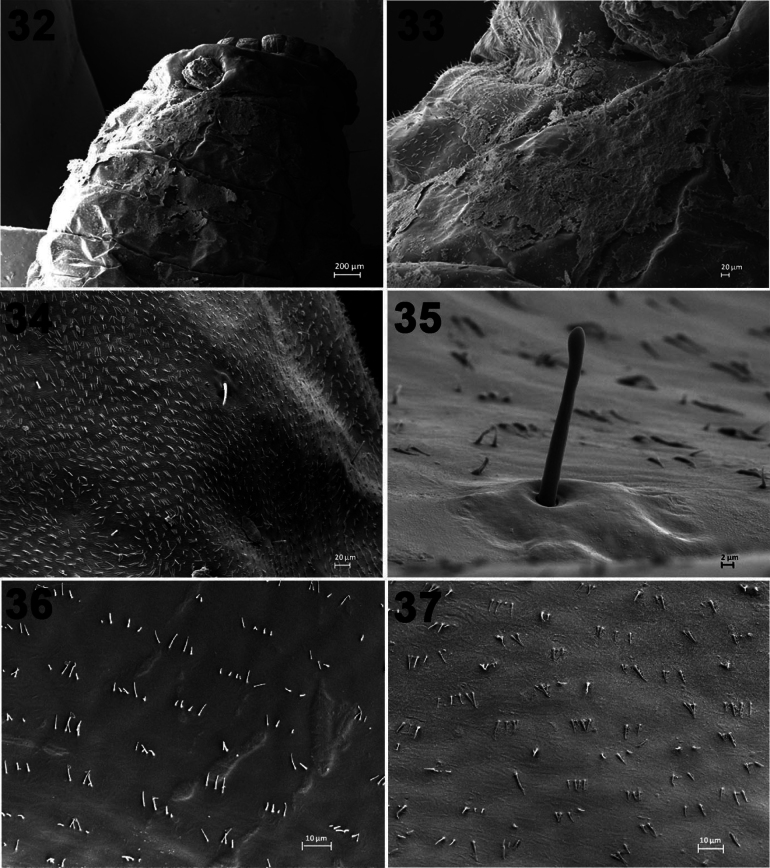
Scanning electron micrographs of larvae of *Podontia quatuordecimpunctata* (Chrysomelidae: Galerucinae: *Blepharida* group) (SEM by M. Schmitt). 32. Specimen L2/1, (instar II) dorso-posterior view showing anus pore in dorsal position; 33. Specimen L2/1, region immediately anterior of the anus, abdominal tergite VIII showing remnants of the fecal shield and – in the left part of the figure – denticles pointing anteriad; 34. Specimen L4/1 (instar IV) lateral region of abdominal tergite VI, showing the densely standing denticles and some setae; 35. Specimen L4/4 (instar IV) a single bristle on the dorsum of the metathorax; 36. Specimen L4/4, tergite VI, denticles pointing anteriad; 37. Specimen L4/4, dorsum of the metathorax, denticles pointing posteriad.

Specialized integumental structures for fecal retention (Figs 32–37). The mature SLB larvae are so large (7–16 mm) that a general overview under SEM is not possible. One instar II larva (specimen L2/1) and one instar IV larva (L4/1), both with fecal remnants (Figs 32, 33), were prepared to show the dorsally pointing anus at the anterior edge of abdominal tergite IX. The anus is a simple pore, and its dorsal opening ensures that feces and fecal fluids are placed directly on the dorsum. The dorsal surface of the larvae is equipped with numerous denticles standing in groups of up to seven in small rows. These denticles are cuticular microstructures arising at the posterior end of the epidermal cells producing the cuticle. They are solid, i.e., they have no lumen, and they do not contain nerve cell extensions (dendrites). The density of these denticles is highest on the lateral regions of the tergites (Fig. 34). Interspersed stand setae (Figs 34, 35) of approximately 10 µm length without a terminal pore. Also, numerous stumps of broken-off setae were found. Two types of setae could be distinguished by their diameter either around 2 or 10 µm.

The denticles stand upright on the sides of the tergites (Fig. 33) or are bent and point in different directions. On the posterior tergites, e.g., on abdominal tergite VIII (Fig. 33) near the anal opening, or on tergite VI (Fig. 33) they point anteriad. On more anterior regions, e.g., on the dorsum of the metathorax (Fig. 34), they are oriented posteriad.

Does the fecal armor protect SLB larvae from insecticides? We address this with trials of insecticide applications to fecal-coated and naked (coat removed) SLB larvae of instars I, II, III, and IV. Results show that the insecticides are effective on both naked and coated instar larvae, with variations in time to reach 100% mortality. Cypermethrin and Imidacloprid exposure produced the same mortality and exposure time in naked and fecal coat instar II larvae. After Imidacloprid treatment, 100% of the cleaned instar II were dead after 5.5 mins and 100% of the fecal-coated larvae died within 6.5 minutes of exposure. After Cypermethrin treatment, 100% of instar II died within 17 h and 40 mins of exposure, so longer than under the Imidacloprid treatment. With coated and naked III–IV larvae, time for 100% mortality was longer, within 17 h 40 m of exposure to Imidacloprid and within 13–17 h after exposure to Cypermethrin. For different larval instars, Carbaryl took 41 h to achieve 100% mortality and Buprofezin (Insect Growth Regulator or IGR) took three days for 100% mortality.

Insecticidal impact on SLB adults. The four insecticides tested were effective (Table 2), resulting in high mortality, but their speed of impact varied from minutes (Cypermethrin, Imidacloprid) to days (Bufropezin, Carbaryl). Availability, cost, speed of degradation in the environment, and site location (e.g., potential pollution to nearby water bodies) are factors to consider in the ultimate selection to control SLB.

## ﻿Discussion

Our study into the fecal armor, its construction, associated morphology, and function complements studies of fecal-constructing behaviors in the other chrysomelid lineages —*Blepharida* group (Vencl and Morton 1998; Huang et al. 2022), Cassidinae (Adam et al. 2022; Chaboo et al. 2023), Criocerinae (Vencl et al. 2004), and Cryptocephalinae (Brown and Funk 2005). The dorsal armor retained directly on the larva’s body is similar in the *Blepharida* group and in Criocerinae.

In wild populations (Figs 2–5) and under experimental conditions (Figs 15–26), SLB larvae build fecal armor with varying moisture levels. Within 8–9 hours, they can cover themselves with a thin coat, and after 24 hours, a thicker one. Over time, feces may clump or fall off.

SEM study revealed that the fecal armor can be kept from falling off the body by denticles. The orientation of these denticles suggests that erect denticles on the lateral parts could keep the fecal cover on the dorsum. Anteriad-pointing denticles on posterior tergites presumably help the feces mass being pushed towards the head by the peristaltic movements of the abdomen. Upright standing or posteriad pointing denticles on abdominal tergites I to V could prevent the feces mass from dropping off or sliding beyond the head. The larvae may perceive the position and weight of the fecal mass by means of the numerous mechanosensitive bristles all over the dorsal surface. Possibly the thinner and the thicker setae are sensitive to different kinds of physical stimuli. The countless stumps found all over the larval body are most probably remnants of setae or chaetae that were broken off when the fecal mass was removed mechanically from the larval surface. These stumps give clear evidence that the setae are mechanoreceptors of the bristle-type (Keil 1997) as they have solid walls and only a central canal and do not show any trace of wall pores. They are mechanosensors, not chemosensors. Given the similarity of fecal coats in the *Blepharida* group and Criocerinae, we suggest that examination of Criocerinae larvae may reveal similar fecal-holding integumental processes.

The insecticidal tests show that the fecal armor slightly delays the insecticides’ toxic effect by a few minutes but does not fully protect them. We suspect that the armor’s moistness or wetness may facilitate adherence of the liquid insecticide, like a sponge. The fecal coat probably delays the potency of the insecticide solution (Table 1). All the insecticides can be used on adults and larvae, but Cypermethrin and Imidacloprid worked faster.

Although the fecal and body fluid coat delays insecticide potency, it does not reduce their efficacy and so the coat does not protect the larvae. Therefore, we recommend rotating these insecticides to prevent the development of resistance in SLB field populations. Our insecticidal results complement our entomopathogenic recommendations (Calcetas et al. 2024) for managing the SLB pest. That study found that fungal entomopathogens were more effective against the SLB pupal and adult stages, less so against larvae due to the protective fecal coats. We suggest using entomopathogenic fungi *Beauveria bassiana* and hand removal when beetle populations are low. Prathapan and Chaboo (2011) also suggested these strategies along with using a parasitoid to manage the population of *Ophrida*, another *Blepharida*-group pest.

The fecal armor and specialized morphology may offer informative characters to support systematics in these taxa. The fecal armor provides limited protection against insecticides compared to *Beauveria bassiana* fungal entomopathogen, which is more effective. To prevent the development of resistance, insecticides should be rotated during high pest populations.

## ﻿Additional information

### Use of AI

No use of AI was reported.

### Author ORCIDs

Orlando A. Calcetas https://orcid.org/0000-0002-8141-9748

Joel L. Adorada https://orcid.org/0000-0002-8335-1388

Michael Schmitt https://orcid.org/0000-0001-7377-3643

Caroline S. Chaboo https://orcid.org/0000-0002-6983-8042

### Data availability

All of the data that support the findings of this study are available in the main text.
